# Submicroscopic deletion of 5q involving tumor suppressor genes (CTNNA1, HSPA9) and copy neutral loss of heterozygosity associated with TET2 and EZH2 mutations in a case of MDS with normal chromosome and FISH results

**DOI:** 10.1186/1755-8166-7-35

**Published:** 2014-05-27

**Authors:** Morteza Hemmat, Weina Chen, Arturo Anguiano, Mohammed El Naggar, Frederick K Racke, Dan Jones, Yongbao Wang, Charles M Strom, Karl Chang, Fatih Z Boyar

**Affiliations:** 1 Cytogenetics Department, Quest Diagnostics Nichols Institute, 33608 Ortega Hwy, 92675 San Juan Capistrano, CA, USA; 2 University of Texas southwestern Medical Center, 5323 Harry Hines Blvd, 75235 Dallas, TX, USA; 3 Quest Diagnostics Nichols Institute, 14225 Newbrook Drive, 20151 Chantilly, VA, USA

**Keywords:** Copy neutral loss of heterozygosity (cnLOH), Uniparental disomy (UPD), MDS, TET2, EZH2, RUNX1, EZH2, ASXL1, CTNNA1, HSPA9

## Abstract

Advances in genome-wide molecular cytogenetics allow identification of novel submicroscopic DNA copy number alterations (aCNAs) and copy-neutral loss of heterozygosity (cnLOH) resulting in homozygosity for known gene mutations in myeloid neoplasms. We describe the use of an oligo-SNP array for genomic profiling of aCNA and cnLOH, together with sequence analysis of recurrently mutated genes, in a patient with myelodysplastic syndrome (MDS) presenting with normal karyotype and FISH results. Oligo-SNP array analysis revealed a hemizygous deletion of 896 kb at chromosome 5q31.2, representing the smallest 5q deletion reported to date. The deletion involved multiple genes, including two tumor suppressor candidate genes (*CTNNA1* and *HSPA9*) that are associated with MDS/AML. The SNP-array study also detected 3 segments of somatic cnLOH: one involved the entire long arm of chromosome 4; the second involved the distal half of the long arm of chromosome 7, and the third encompassed the entire chromosome 22 (UPD 22). Sequence analysis revealed mutations in *TET2* (4q), *EZH2* (7q), *ASXL1* (20q11.21), and *RUNX1* (21q22.3). Coincidently, *TET2* and *EZH2* were located at segments of cnLOH resulting in their homozygosity. Loss of heterozygosity affecting these two chromosomes and mutations in *TET2* and *EZH2* are indicative of a myelodysplastic syndrome with a poor prognosis. Deletion of the tumor suppressor genes *CTNNA1* and *HSPA9* is also likely to contribute to a poor prognosis. Furthermore, the original cnLOHs in multiple chromosomes and additional cnLOH 14q in the follow-up study suggest genetic evolution of the disease and poor prognosis. This study attests to the fact that some patients with a myelodysplastic syndrome who exhibit a normal karyotype may have underlying genetic abnormalities detectable by chromosomal microarray and/or targeted mutation analyses.

## Background

Recent advances in genome-wide molecular cytogenetics allow the identification of novel molecular abnormalities [1-8]. Emerging data demonstrate that myelodysplastic syndrome (MDS) exhibits abundant CNAs and cnLOH, often in the setting of a normal karyotype [9-11]. Loss of heterozygosity (LOH) is an indicator of neoplastic evolution and disease progression [12,13]. Copy-neutral LOH (cnLOH) arises either via a hemizygous deletion in one homolog and duplication of the other, or uniparental disomy (UPD). Both types of somatic LOH have been observed in studies of various cancer types and may explain some of the mechanisms by which tumor suppressor genes (TSGs) are inactivated or activating mutations in oncogenes are duplicated. Acquired UPD (aUPD) is now understood to be common in oncogenesis and appears to be a mechanism to increase the allelic burden of the mutated genes [14-24]. Thus, it is important to know the regions of cnLOH to determine new regions containing potential mutational targets affecting disease pathogenesis and treatment outcome [25,26].

Application of SNP-array technology has led to the identification of recurrent regions of cnLOH in a majority of the chromosomes [4,13,25,27-30] and recurrent pathogenic mutations. These findings have greatly advanced our understanding of the molecular mechanisms of cancer evolution and have led to the development of therapeutics and diagnostic tests. In this respect, mutation analysis helps further stratify neoplasms and their treatment outcome [31-34].

In this study, we used SNP array-based genomic profiling to detect acquired copy number alterations (aCNA) and cnLOHs, together with sequence analysis of genes commonly mutated in MDS, in a patient with normal chromosome and MDS FISH (fluorescence in situ hybridization) panel results.

## Clinical presentation

### Morphologic and immunophenotypic findings

**Figure 1:**
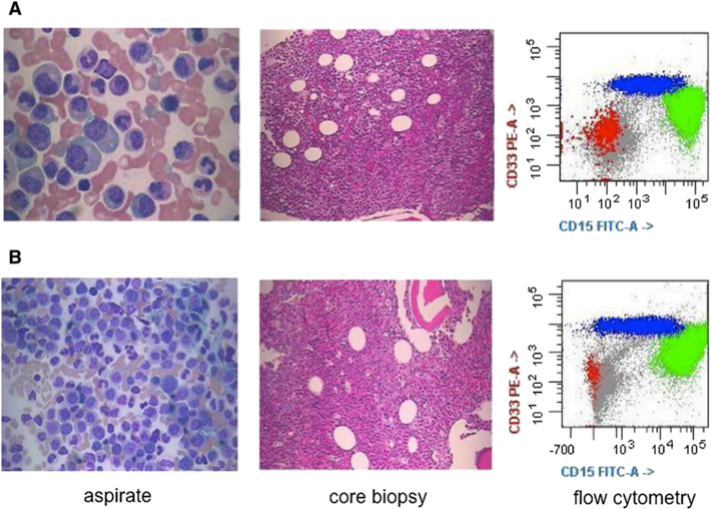
**Morphologic and immunophenotypic findings. A)** At presentation, there was granulocytic predominance with progressive maturation and no significant increase in blasts or monocytes. The core biopsy shows hypercellular bone marrow with left-shifted granulocytic predominance. Flow cytometry reveals granulocytic predominance (in green) but no significant increase in monocytes (in blue) or myeloblasts (in red). **B)** At follow-up, the morphologic and immunophenotypic findings are similar to those at presentation.

An 88-year-old woman presented with leukocytosis (16.2×10^9^/L) including minimal monocytosis (1.0×10^9^/L), mild anemia (HGB 105 g/L), and moderate thrombocytopenia (79×10^9^/L). Bone marrow morphologic and flow cytometric examinations revealed hypercellular bone marrow with granulocytic predominance with left-shifted and unusual maturation, and occasional atypical granulocytes and megakaryocytes, but no significant increase in monocytes or myeloblasts (Figure 1). A myeloid neoplasm positive for CD13 and CD33 was diagnosed, best classified as MDS, refractory cytopenia with multilineage dysplasia. Data on therapeutic interventions were not available for this study. At the 5-month follow-up, the overall morphologic and immunophenotypic findings were similar to those at initial presentation.

### Chromosome and FISH study

Bone marrow cells were cultivated for 24 and 48 hours in RPMI 1640 or Marrow Max Medium with 10% fetal calf serum (Life Technologies, Grand Island, NY 14072, USA). In total, 20 mitoses were analyzed according to the ISCN (International System for Human Cytogenetic Nomenclature), with a resolution of 300 bands per haploid karyotype. FISH analyses using a panel of MDS probes (-5/5q-, -7/7q-, +8 and 20q-; Vysis, Downers Grove, IL, USA) and BlueGnome probes RP11-114B12 (Illumina, San Diego, CA, USA) for the deleted region 5q31.2 were performed on interphase cells according to the manufacturer’s protocol. Subsequently, 200 cells were examined carefully.

### Oligo SNP array

Microdeletion/microduplication screening was performed using an SNP-array platform (CytoScan HD SNP array; Affymetrix, Santa Clara, CA), following the manufacturer’s instructions. The CytoScan HD array has 2.67 million probes, including 1.9 million copy number probes and 0.75 million SNP probes. Array data were analyzed using the Chromosome Analysis Suite (ChAS) software v 2.0 (Affymetrix).

### Mutation analysis

Genomic DNA was also tested for mutations in 19 genes that are recurrently mutated in myeloid neoplasms, including ASXL1, EZH2, RUNX1, IDH1, IDH2, KRAS, NRAS and TET2. Sequencing was performed using a TruSeq custom amplicon assay on the MiSeq sequencing platform (Illumina, Hayward, CA). Analysis was performed using SeqPilot software (JSI Medical Systems, Costa Mesa, CA). The assay had sufficient read depth to provide a minimum sensitivity of 5% to 10% for mutation detection.

## Results and discussion

**Figure 2:**
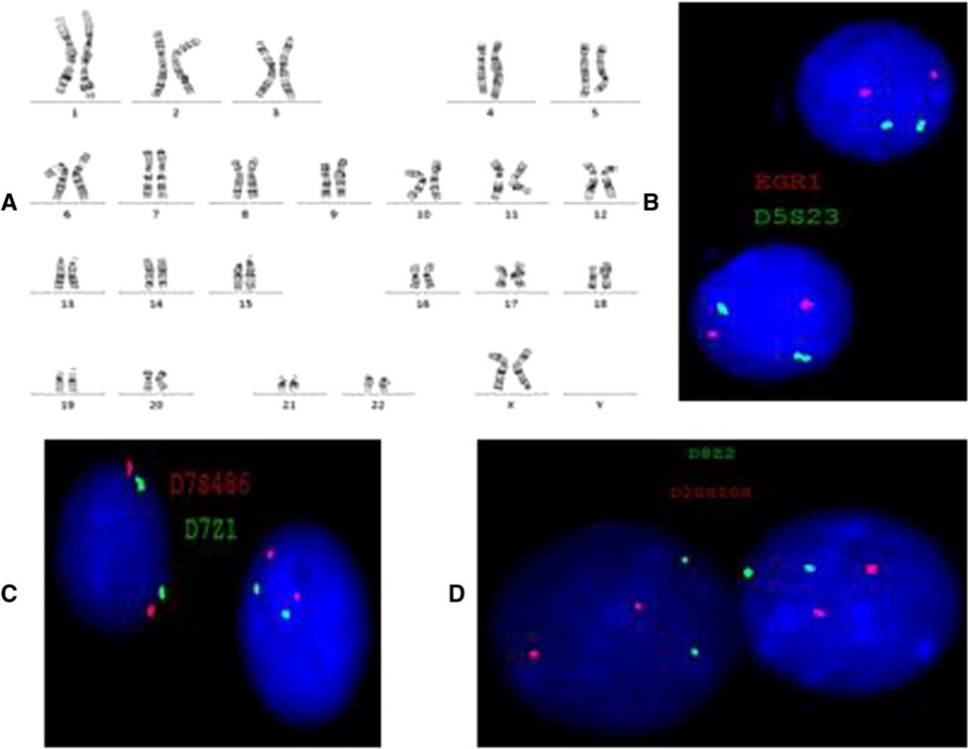
**Routine cytogenetic and fluorescence insitu hybridization (FISH) results. A)** G-banded chromosome analysis shows a normal female karyotype. **B-D)** FISH analyses show normal hybridization with an MDS panel using probes for EGR1 (5q31, red signal) and its control probe D5S23 (5p15.2, green signal) in **B**; D7S486 (7q31, red signal) and its control probe D7Z1 (7centromere, green signal) in **C**; CEP8 (green signal) for chromosome 8 centromere and D20S108 (20q12, red signal) in **D**.

**Figure 3:**
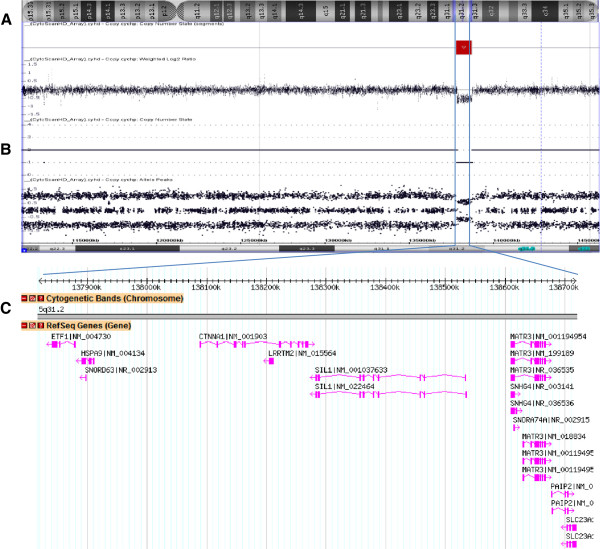
**5q31.2 deletion. A)** Chromosome 5 with deletion at q31.2. **B)** SNP-array results, including the weighted log2 ratio, copy number state, and allele peaks at the deleted region. **C)** Database of genomic variants showing an 896-kb deletion in the short arm of chromosome 5 within band q31.2 (position 137,821,899 to 138,718,504), including the *CTNNA1* and *HSPA9* genes.

During the initial evaluation of the patient, cytogenetic analysis revealed a normal karyotype and FISH studies were negative for aCNAs commonly seen in MDS (MDS panel) (Figure 2A-D). Microarray analysis revealed a microdeletion of approximately 896 kb at the 5q31.2 chromosomal region and three segments of somatic cnLOH for the entire long arm of chromosome 4 (136 Mb), the distal half of the long arm of chromosome 7 (50 Mb), and the entire chromosome 22 (31 Mb). The microdeletion at 5q31.2 extended from 137,821,899 to 138,718,504 bp (UCSC genome Browser; http://genome.ucsc.edu/; hg19 release) and included the *ETF1, HSPA9, SNORD63, CTNNA1, LRRTM2, SIL1, SNHG4, MATR3, SNORA74A, PAIP2*, and *SLC23A1* genes (Figure 3).

**Figure 4:**
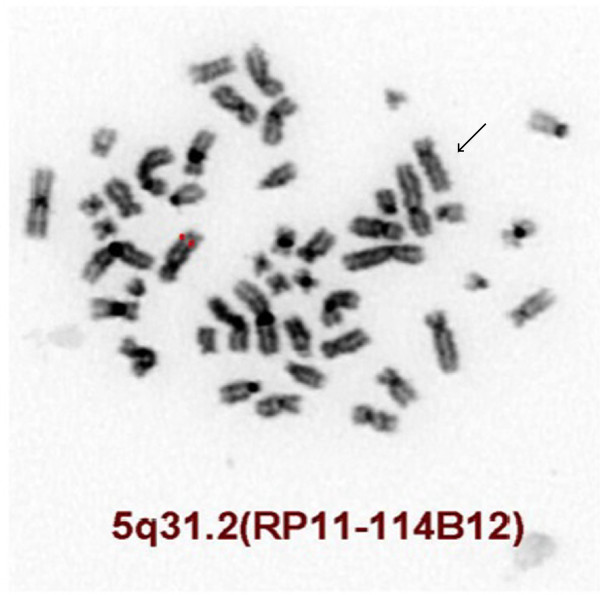
**FISH inverted DAPI image showing deletion of the *CTNNA1 *gene using the BlueGnome FISH probe RP11-114B12 (5q31.2, red signal).** The deleted chromosome 5 is indicated by an arrow.

Of the genes deleted due to this microdeletion, two are tumor suppressor candidate genes associated with MDS/AML: *CTNNA1*, which encodes alpha-1 catenin, and *HSPA9*, which encodes heat-shock 70-KD protein 9 (mortalin) [35]. Deletion of *CTNNA1* was confirmed by applying BlueGnome FISH probes (RP11-114B12) (Figure 4). Alpha-catenins such as that encoded by *CTNNA1* are essential for the regulation of cell-cell and cell-matrix interactions in tissues [36]. Loss of expression of the *CTNNA1* tumor suppressor gene in hematopoietic stem cells may provide a growth advantage that contributes to human MDS/AML with 5q deletion [37]. Furthermore, loss of the *CTNNA1* expression has been associated with leukemia progression or transformation of MDS to AML [38]. The *HSPA9* gene is also located at the 5q31.2 region frequently deleted in MDS/AML, making it a candidate tumor suppressor gene; this is consistent with the biological function of its murine homologue. Human mortalin (encoded by *HSPA9*) was originally identified by its close homology to murine mortalins, which play important roles in cellular senescence [39]. The *HSPA9* gene is a novel negative regulator of Raf/MEK/ERK pathway that may be a potential therapeutic target [40].

**Figure 5:**
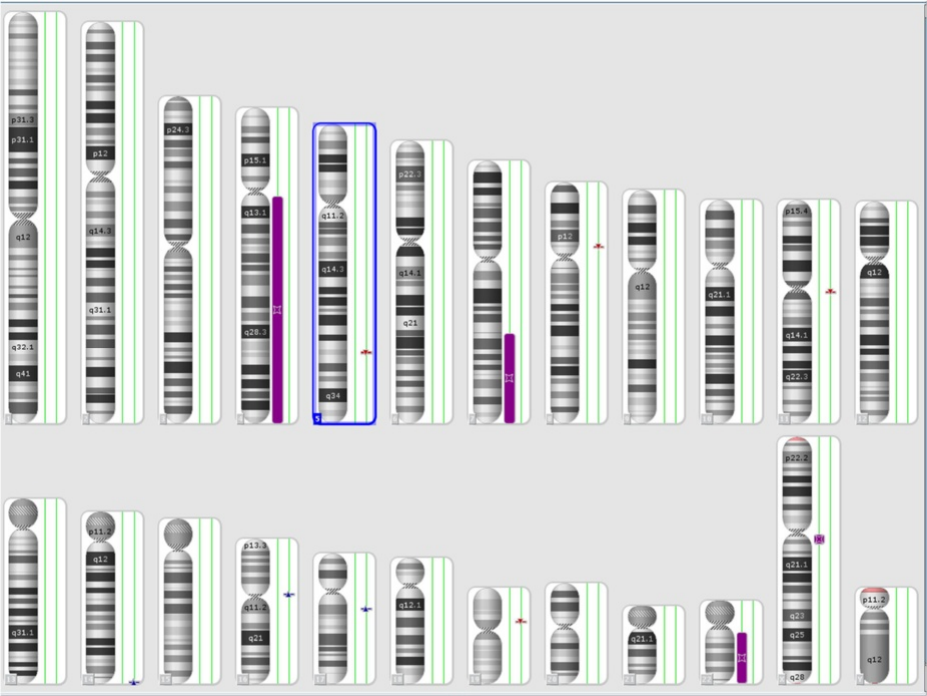
SNP-array results at diagnosis showing loss of heterozygosity (LOH) at the long arm of chromosomes 4, 7, and 22 as purple bars next to the corresponding chromosomes.

**Figure 6:**
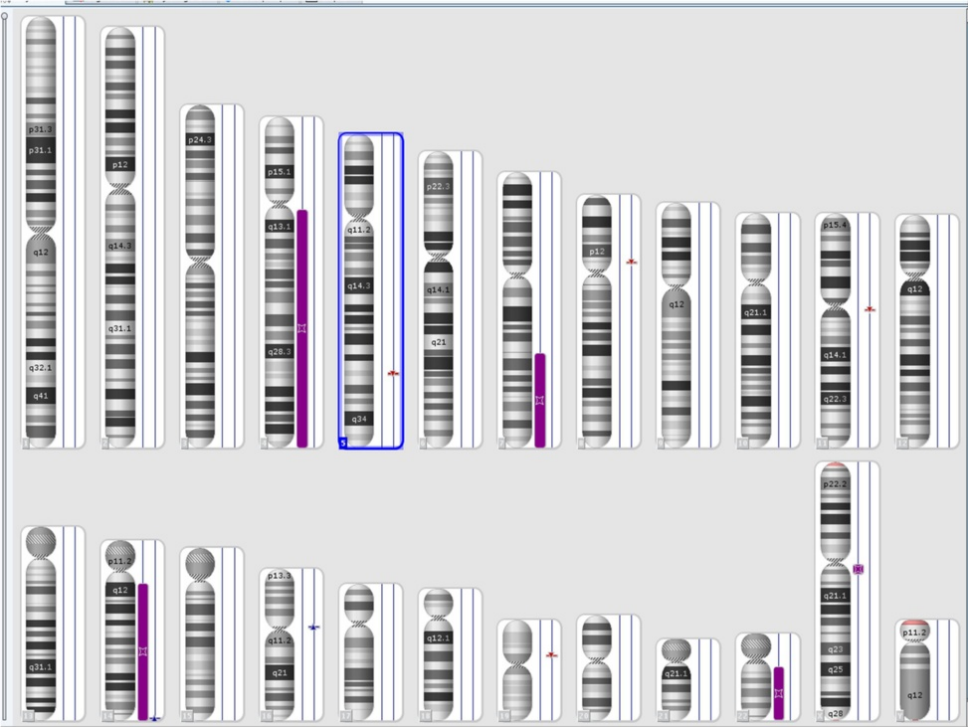
**SNP-array results at the follow-up study showing loss of heterozygosity (LOH) of chromosome 14, in addition to the LOH chromosomes 4, 7, and 22 found at initial diagnosis.** LOH is shown as purple bar next to the corresponding chromosome.

The combined size of cnLOHs spanning at least 10 Mb across the genome was approximately 217.6 Mb (Figure 5). These were detected at the long arm of chromosomes 4, 7, and 22. The cnLOH might result from mitotic recombination or nondisjunction which leads to segmental or whole chromosomal UPD, respectively [13]. Acquired UPD (cnLOH) at diagnosis in our case is indicative of neoplastic evolution [12,13]. An additional UPD was identified at 14q in the follow-up study 5 months later, confirming the genetic progression of disease (Figure 6).

Recent investigations have indicated that cnLOH can be responsible for homozygosity of mutations in critical genes within the region. Reduction to homozygosity as a consequence of cnLOH was initially thought to be a mechanism for only inactivation of tumor suppressor genes [14,41,42]; however, identification of cnLOH in leukemia has shown that oncogeneic mutations are also targeted [13,16,17].

Mutation analysis of 19 MDS-associated genes revealed an *ASXL1* frameshift/stop mutation (Y591*, 41% of reads), an *EZH2* point mutation (R690H, 85%), two *RUNX1* frameshift/stop mutations (c.474dupT, 19% and c.424_425ins11bp, 7%), and two *TET2* frameshift/stop mutations (c.1510_1513delAAAA, 77% and R1465*, 10%). These mutated genes are located on chromosomes 20, 7, 21, and 4, respectively. Recent studies have shown that *TET2* mutations are present in up to 30% of MDS/MPN cases, with mutations in *ASXL1, EZH2,* and *RUNX1* also commonly reported [43]. The finding of mutations in all four of these myeloid regulatory genes suggests that they play a critical role in the pathogenesis of this case and demonstrate that mutation analysis is useful in cytogenetically normal myeloid disorders [43].

The cnLOH affecting chromosome 7q and homozygous *EZH2* mutation have been reported in 10% of AML and MDS cases. They have been associated with a poor prognosis [9,26,44] and clonal evolution [45,46], supporting the possible role of *EZH2* as a tumor suppressor gene for myeloid malignancies.

In contrast to the mutated *TET2* and *EZH2* genes, no LOH was found for the other two mutated genes (*RUNX1* and *ASXL1*). *RUNX1* mutations have been proposed as clinically useful biomarkers to follow disease progression from MDS to AML as well as to monitor minimal residual disease (MRD) [47]. Moreover, *RUNX1* mutations were demonstrated to be frequent in *de novo* AML with non-complex karyotypes and conferred an unfavorable prognosis [48] explained by an association with resistance to chemotherapy [49].

Mutations in *ASXL1* have been identified in MDS, AML, chronic myeloid leukemia, chronic myelomonocytic leukemia (CMML), and juvenile myelomonocytic leukemia [50-54], and act as a tumor suppressor in myeloid malignancies [50]. Mutations in *ASXL1*, *TET2*, and *EZH2* have been found in 41% of MDS cases in a Chinese population [44], similar to the data reported in patients of European decent with MDS [21,23,24,55,56].

## Conclusion

In conclusion, our study identified four large cnLOH and a microdeletion at 5q31 harboring two tumor suppressor genes (*CTNNA1* and *HSPA9*) in an MDS case with an apparently normal karyotype. The regions of cnLOH at chromosomes 4, 7, 14, and 22 confirm and extend previous studies, supporting that cnLOHs in myeloid disorders are common and nonrandom. Moreover, the presence of an additional aUPD 14q at follow up, along with persistence of the 5q31 microdeletion and all cnLOHs detected at presentation, indicates genetic progression of the disease. The deletion of tumor suppressor genes *CTNNA1* and *HSPA9*, along with mutation of candidate myeloid regulatory genes *TET2* and *EZH2*, supports the diagnosis of MDS and likely portends a poor prognosis.

This study attests to the fact that some patients with a myelodysplastic syndrome exhibiting a normal karyotype may have underlying genetic abnormalities detectable by chromosomal microarray and/or targeted mutation analyses. Further genomic and molecular studies on a series of patients with MDS may yield information on how to stratify this category of disease to seek further molecular definition.

## Ethical approval and consent

These studies were performed on anonymized samples received in the clinical laboratory and thus were exempted from the requirement for consent by an opinion for the Western Institutional Review Board.

## Abbreviations

aCNA: Acquired copy number alteration; cnLOH: Loss of heterozygosity; aUPD: Acquired uniparental disomy.

## Competing interests

The authors declare that they have no competing interests.

## Authors’ contributions

MH, First author; performed chromosome, FISH, and microarray analysis, interpretation of the results, drafting and finalization of the manuscript. WC performed the morphology and immunophenotypic analysis. DJ and YW performed the genomic sequencing. FR performed the comprehensive review of the clinical and laboratory findings. AA reviewed and reported the SNP-array analyses. MEN reviewed the manuscript. FZB reviewed and finalized the manuscript. All authors read and approved the final manuscript.

## References

[B1] Mullighan CG Goorha S Radtke I Miller CB Coustan-Smith E Dalton JD Girtman K Mathew S Ma J Pounds SB Su X Pui CH Relling MV Evans WE Shurtleff SA Downing JR Genome-wide analysis of genetic alterations in acute lymphoblastic leukemia Nature 2007 446 758 764 10.1038/nature05690 17344859

[B2] Suela J Alvarez S Cifuentes F Largo C Ferreira BI Blesa D Ardanaz M Garcia R Marquez JA Odero MD Calasanz MJ Cigudosa JC DNA profiling analysis of 100 consecutive de novo acute myeloid leukemia cases reveals patterns of genomic instability that affect all cytogenetic risk groups Leukemia 2007 21 1224 1231 10.1038/sj.leu.2404653 17377590

[B3] Tyyba kinoja A Elonen E Piippo K Porkka K Knuutila S Oligonucleotide array-CGH reveals cryptic gene copy number alterations in karyotypically normal acute myeloid leukemia Leukemia 2007 21 571 574 10.1038/sj.leu.2404543 17268525

[B4] Gupta M Raghavan M Gale RE Chelala C Allen C Molloy G Chaplin T Linch DC Cazier JB Young BD Novel regions of acquired uniparental disomy discovered in acute myeloid leukemia Genes Chromosomes Cancer 2008 47 729 739 10.1002/gcc.20573 18506749

[B5] Kawamata N Ogawa S Zimmermann M Kato M Sanada M Hemminki K Yamatomo G Nannya Y Koehler R Flohr T Miller CW Harbott J Ludwig WD Stanulla M Schrappe M Bartram CR Koeffler HP Molecular allelokaryotyping of pediatric acute lymphoblastic leukemias by high-resolution single nucleotide polymorphism oligonucleotide genomic microarray Blood 2008 111 2 776 84 10.1182/blood-2007-05-088310 17890455 PMC2200831

[B6] Akagi T Ogawa S Dugas M Kawamata N Yamamoto G Nannya Y Sanada Miller CW Yung A Schnittger S Haferlach T Haferlach C Koeffler HP Frequent genomic abnormalities in acute myeloid leukemia/myelodysplastic syndrome with normal karyotype Haematologica 2009 94 213 223 10.3324/haematol.13024 19144660 PMC2635399

[B7] Maciejewski JP Tiu RV O’Keefe C Application of array based whole genome scanning technologies as a cytogenetic tool in haematological malignancies Br J Hematol 2009 146 479 488 10.1111/j.1365-2141.2009.07757.x 19563474

[B8] Tiu RV Gondek LP O’Keefe CL Huh J Sekeres MA Elson P McDevitt MA Wang XF Levis MJ Karp JE Advani AS Maciejewski JP New lesions detected by single nucleotide polymorphism array-based chromosomal analysis have important clinical impact in acute myeloid leukemia J Clin Oncol 2009 27 5219 5226 10.1200/JCO.2009.21.9840 19770377 PMC2773477

[B9] Heinrichs S Kulkarni RV Bueso-Ramos CE Levine RL Loh ML Li C Neuberg D Kornblau SM Issa JP Gilliland DG Garcia-Manero G Kantarjian HM Estey EH Look AT Accurate detection of uniparental disomy and microdeletions by SNP array analysis in myelodysplastic syndromes with normal cytogenetics Leukemia 2009 23 9 1605 13 10.1038/leu.2009.82 19387468 PMC2950785

[B10] Thiel A1 Beier M Ingenhag D Servan K Hein M Moeller V Betz B Hildebrandt B Evers C Germing U Royer-Pokora B Comprehensive array CGH of normal karyotype myelodysplastic syndromes reveals hidden recurrent and individual genomic copy number alterations with prognostic relevance Leukemia 2011 25 387 399 10.1038/leu.2010.293 21274003

[B11] Tiu RV1 Gondek LP O’Keefe CL Elson P Huh J Mohamedali A Kulasekararaj A Advani AS Paquette R List AF Sekeres MA McDevitt MA Mufti GJ Maciejewski JP Prognostic impact of SNP array karyotyping in myelodysplastic syndromes and related myeloid malignancies Blood 2011 117 17 4552 60 10.1182/blood-2010-07-295857 21285439 PMC3099573

[B12] Andersen CL Wiuf C Kruhøffer M Korsgaard M Laurberg S Ørntoft TF Frequent occurrence of uniparental disomy in colorectal cancer Carcinogenesis 2007 28 1 38 48 10.1093/carcin/bgl086 16774939

[B13] Raghavan M Smith LL Lillington DM Chaplin T Kakkas I Molloy G Chelala C Cazier JB Cavenagh JD Fitzgibbon J Lister TA Young BD Segmental uniparental disomy is a commonly acquired genetic event in relapsed acute myeloid leukemia Blood 2008 112 3 814 21 10.1182/blood-2008-01-132431 18490517

[B14] Flotho C Steinemann D Mullighan CG Neale G Mayer K Kratz CP Schlegelberger B Downing JR Niemeyer CM Genome-wide single-nucleotide polymorphism analysis in juvenile myelomonocytic leukemia identifies uniparental disomy surrounding the NF1 locus in cases associated with neurofibromatosis but not in cases with mutant RAS or PTPN11 Oncogene 2007 26 39 5816 21 10.1038/sj.onc.1210361 17353900

[B15] Fitzgibbon J Smith LL Raghavan M Smith ML Debernardi S Skoulakis S Lillington D Lister TA Young BD Association between acquired uniparental disomy and homozygous gene mutation in acute myeloid leukemias Cancer Res 2005 65 9152 9154 10.1158/0008-5472.CAN-05-2017 16230371

[B16] Kralovics R Passamonti F Buser AS Teo SS Tiedt R Passweg JR Tichelli A Cazzola M Skoda RC A gain-of-function mutation of JAK2 in myeloproliferative disorders N Engl J Med 2005 352 17 1779 90 10.1056/NEJMoa051113 15858187

[B17] Kralovics R Guan Y Prchal JT Acquired uniparental disomy of chromosome 9p is a frequent stem cell defect in polycythemia vera Exp Hematol 2002 30 3 229 36 10.1016/S0301-472X(01)00789-5 11882360

[B18] Grand FH Hidalgo-Curtis CE Ernst T Zoi K Zoi C McGuire C Kreil S Jones A Score J Metzgeroth G Oscier D Hall A Brandts C Serve H Reiter A Chase AJ Cross NC Frequent CBL mutations associated with 11q acquired uniparental disomy in myeloproliferative neoplasms Blood 2009 113 24 6182 92 10.1182/blood-2008-12-194548 19387008

[B19] Sanada M Suzuki T Shih LY Otsu M Kato M Yamazaki S Tamura A Honda H Sakata-Yanagimoto M Kumano K Oda H Yamagata T Takita J Gotoh N Nakazaki K Kawamata N Onodera M Nobuyoshi M Hayashi Y Harada H Kurokawa M Chiba S Mori H Ozawa K Omine M Hirai H Nakauchi H Koeffler HP Ogawa S Gain-of-function of mutated C-CBL tumour suppressor in myeloid neoplasms Nature 2009 460 7257 904 8 10.1038/nature08240 19620960

[B20] Dunbar AJ Gondek LP O’Keefe CL Makishima H Rataul MS Szpurka H Sekeres MA Wang XF McDevitt MA Maciejewski JP 250 K single nucleotide polymorphism array karyotyping identifies acquired uniparental disomy and homozygous mutations, including novel missense substitutions of c-Cbl, in myeloid malignancies Cancer Res 2008 68 24 10349 57 10.1158/0008-5472.CAN-08-2754 19074904 PMC2668538

[B21] Ernst T Chase AJ Score J Hidalgo-Curtis CE Bryant C Jones AV Waghorn K Zoi K Ross FM Reiter A Hochhaus A Drexler HG Duncombe A Cervantes F Oscier D Boultwood J Grand FH Cross NC Inactivating mutations of the histone methyltransferase gene EZH2 in myeloid disorders Nat Genet 2010 42 8 722 6 10.1038/ng.621 20601953

[B22] Langemeijer SM Kuiper RP Berends M Knops R Aslanyan MG Massop M Stevens-Linders E van Hoogen P van Kessel AG Raymakers RA Kamping EJ Verhoef GE Verburgh E Hagemeijer A Vandenberghe P de Witte T van der Reijden BA Jansen JH Acquired mutations in TET2 are common in myelodysplastic syndromes Nat Genet 2009 41 7 838 42 10.1038/ng.391 19483684

[B23] Nikoloski G Langemeijer SM Kuiper RP Knops R Massop M Tönnissen ER van der Heijden A Scheele TN Vandenberghe P de Witte T van der Reijden BA Jansen JH Somatic mutations of the histone methyltransferase gene EZH2 in myelodysplastic syndromes Nat Genet 2010 42 8 665 7 10.1038/ng.620 20601954

[B24] Delhommeau F Dupont S Della Valle V James C Trannoy S Massé A Kosmider O Le Couedic JP Robert F Alberdi A Lécluse Y Plo I Dreyfus FJ Marzac C Casadevall N Lacombe C Romana SP Dessen P Soulier J Viguié F Fontenay M Vainchenker W Bernard OA Mutation in TET2 in myeloid cancers N Engl J Med 2009 360 22 2289 301 10.1056/NEJMoa0810069 19474426

[B25] Mohamedali A Gäken J Twine NA Ingram W Westwood N Lea NC Hayden J Donaldson N Aul C Gattermann N Giagounidis A Germing U List AF Mufti GJ Prevalence and prognostic significance of allelic imbalance by single-nucleotide polymorphism analysis in low-risk myelodysplastic syndromes Blood 2007 110 9 3365 73 10.1182/blood-2007-03-079673 17634407

[B26] Gondek LP Tiu R O'Keefe CL Sekeres MA Thiel KS Maciejewski JP Chromosomal lesions and uniparental disomy detected by SNP arrays in MDS Blood 2008 111 1534 42 17954704 10.1182/blood-2007-05-092304 PMC2214746

[B27] Gorletta TA Gasparini P D’Elios MM Trubia M Pelicci PG Di Fiore PP Frequent loss of heterozygosity without loss of genetic material in acute myeloid leukemia with a normal karyotype Genes Chromosomes Cancer 2005 44 334 337 10.1002/gcc.20234 16015648

[B28] Raghavan M Lillington DM Skoulakis S Debernardi S Chaplin T Foot NJ Lister TA Young BD Genome-wide single nucleotide polymorphism analysis reveals frequent partial uniparental disomy due to somatic recombination in acute myeloid leukemias Cancer Res 2005 65 375 378 15695375

[B29] Fitzgibbon J Smith LL Raghavan M Smith ML Debernardi S Skoulakis S Lillington D Lister TA Young BD Association between acquired uniparental disomy and homozygous gene mutation in acute myeloid leukemias Cancer Res 2005 65 9152 9154 10.1158/0008-5472.CAN-05-2017 16230371

[B30] Gondek LP Dunbar AJ Szpurka H McDevitt MA Maciejewski JP SNP array karyotyping allows for the detection of uniparental disomy and cryptic chromosomal abnormalities in MDS/MPD-U and MPD PLoS One 2007 2 11 e122 10.1371/journal.pone.0001225 PMC2075364 18030353

[B31] Kottaridis PD Gale RE Frew ME Harrison G Langabeer SE Belton AA Walker H Wheatley K Bowen DT Burnett AK Goldstone AH Linch DC The presence of a FLT3 internal tandem duplication in patients with acute myeloid leukemia (AML) adds important prognostic information to cytogenetic risk group and response to the first cycle of chemotherapy: Analysis of 854 patients from the United Kingdom medical research council. AML 10 and 12 trials Blood 2001 98 1752 1759 10.1182/blood.V98.6.1752 11535508

[B32] Falini B Mecucci C Tiacci E Alcalay M Rosati R Pasqualucci L La Starza R Diverio D Colombo E Santucci A Bigerna B Pacini R Pucciarini A Liso A Vignetti M Fazi P Meani N Pettirossi V Saglio G Mandelli F Lo-Coco F Pelicci PG Martelli MF Cytoplasmic nucleophosmin in acute myelogenous leukemia with a normal karyotype N Engl J Med 2005 352 254 266 10.1056/NEJMoa041974 15659725

[B33] Baldus CD Mrozek K Marcucci G Bloomfield CD Clinical outcome of de novo acute myeloid leukaemia patients with normal cytogenetics is affected by molecular genetic alterations: a concise review Br J Haematol 2007 137 387 400 10.1111/j.1365-2141.2007.06566.x 17488484

[B34] Mead AJ Linch DC Hills RK Wheatley K Burnett AK Gale RE FLT3 tyrosine kinase domain mutations are biologically distinct from and have a significantly more favorable prognosis than FLT3 internal tandem duplications in patients with acute myeloid leukemia Blood 2007 110 1262 1270 10.1182/blood-2006-04-015826 17456725

[B35] Horrigan SK Arbieva ZH Xie HY Kravarusic J Fulton NC Naik H Le TT Westbrook CA Delineation of a minimal interval and identification of 9 candidates for a tumor suppressor gene in malignant myeloid disorders on 5q31 Blood 2000 95 7 2372 7 10733509

[B36] Kask M1 Pruunsild P Timmusk T Bidirectional transcription from human LRRTM2/CTNNA1 and LRRTM1/CTNNA2 gene loci leads to expression of N-terminally truncated CTNNA1 and CTNNA2 isoforms Biochem Biophys Res Commun 2011 411 1 56 61 10.1016/j.bbrc.2011.06.085 21708131

[B37] Liu TX Becker MW Jelinek J Wu WS Deng M Mikhalkevich N Hsu K Bloomfield CD Stone RM DeAngelo DJ Galinsky IA Issa JP Clarke MF Look AT Chromosome 5q deletion and epigenetic suppression of the gene encoding alpha-catenin (CTNNA1) in myeloid cell transformation Nat Med 2007 13 1 78 83 10.1038/nm1512 17159988

[B38] Ye Y McDevitt MA Guo M Zhang W Galm O Gore SD Karp JE Maciejewski JP Kowalski J Tsai HL Gondek LP Tsai HC Wang X Hooker C Smith BD Carraway HE Herman JG Progressive chromatin repression and promoter methylation of CTNNA1 associated with advanced myeloid malignancies Cancer Res 2009 69 21 8482 90 10.1158/0008-5472.CAN-09-1153 19826047 PMC3081599

[B39] Xie H Hu Z Chyna B Horrigan SK Westbrook CA Human mortalin (HSPA9): a candidate for the myeloid leukemia tumor suppressor gene on 5q31 Leukemia 2000 14 12 2128 34 10.1038/sj.leu.2401935 11187902

[B40] Wu PK Hong SK Veeranki S Karkhanis M Starenki D Plaza JA Park JI A mortalin/HSPA9-mediated switch in tumor-suppressive signaling of Raf/MEK/extracellular signal-regulated kinase Mol Cell Biol 2013 33 20 4051 67 10.1128/MCB.00021-13 23959801 PMC3811686

[B41] Cavenee WK Dryja TP Phillips RA Benedict WF Godbout R Gallie BL Murphree AL Strong LC White RL Expression of recessive alleles by chromosomal mechanisms in retinoblastoma Nature 1983 305 5937 779 84 10.1038/305779a0 6633649

[B42] Fearon ER Vogelstein B Feinberg AP Somatic deletion and duplication of genes on chromosome 11 in Wilms’ tumours Nature 1984 309 176 8 10.1038/309176a0 6325939

[B43] Jankowska AM Szpurka H Tiu RV Makishima H Afable M Huh J O’Keefe CL Ganetzky R McDevitt MA Maciejewski JP Loss of heterozygosity 4q24 and TET2 mutations associated with myelodysplastic/myeloproliferative neoplasms Blood 2009 113 25 6403 10 10.1182/blood-2009-02-205690 19372255 PMC2710933

[B44] Wang J Ai X Gale RP Xu Z Qin T Fang L Zhang H Pan L Hu N Zhang Y Xiao Z TET2, ASXL1 and EZH2 mutations in Chinese with myelodysplastic syndromes Leuk Res 2013 37 3 305 11 10.1016/j.leukres.2012.10.004 23099237

[B45] Jerez A1 Sugimoto Y Makishima H Verma A Jankowska AM Przychodzen B Visconte V Tiu RV O’Keefe CL Mohamedali AM Kulasekararaj AG Pellagatti A McGraw K Muramatsu H Moliterno AR Sekeres MA McDevitt MA Kojima S List A Boultwood J Mufti GJ Maciejewski JP Loss of heterozygosity in 7q myeloid disorders: clinical associations and genomic pathogenesis Blood 2012 119 25 6109 17 10.1182/blood-2011-12-397620 22553315 PMC3383019

[B46] Khan SN Jankowska AM Mahfouz R Dunbar AJ Sugimoto Y Hosono N Hu Z Cheriyath V Vatolin S Przychodzen B Reu FJ Saunthararajah Y O’Keefe C Sekeres MA List AF Moliterno AR McDevitt MA Maciejewski JP Makishima H Multiple mechanisms deregulate EZH2 and histone H3 lysine 27 epigenetic changes in myeloid malignancies Leukemia 2013 27 6 1301 9 10.1038/leu.2013.80 23486531

[B47] Dicker F Haferlach C Sundermann J Wendland N Weiss T Kern W Haferlach T Schnittger S Mutation analysis for RUNX1, MLL-PTD, FLT3-ITD, NPM1 and NRAS in 269 patients with MDS or secondary AML Leukemia 2010 24 1528 1532 10.1038/leu.2010.124 20520634

[B48] Schnittger S Dicker F Kern W Wendland N Sundermann J Alpermann T Alpermann T Haferlach C Haferlach T RUNX1 mutations are frequent in de novo AML with noncomplex karyotype and confer an unfavorable prognosis Blood 2011 117 2348 2357 10.1182/blood-2009-11-255976 21148331

[B49] Gaidzik VI Bullinger L Schlenk RF Zimmermann AS Rock J Paschka P Corbacioglu A Krauter J Schlegelberger B Ganser A Späth D Kündgen A Schmidt-Wolf IG Götze K Nachbaur D Pfreundschuh M Horst HA Döhner H Döhner K RUNX1 mutations in acute myeloid leukemia: results from a comprehensive genetic and clinical analysis from the AML study group J Clin Oncol 2011 29 1364 1372 10.1200/JCO.2010.30.7926 21343560

[B50] Gelsi-Boyer V Trouplin V Adélaïde J Bonansea J Cervera N Carbuccia N Lagarde A Prebet T Nezri M Sainty D Olschwang S Xerri L Chaffanet M Mozziconacci MJ Vey N Birnbaum D Mutations of polycomb-associated gene ASXL1 in myelodysplastic syndromes and chronic myelomonocytic leukaemia Br J Haematol 2009 145 6 788 800 10.1111/j.1365-2141.2009.07697.x 19388938

[B51] chou WC Hou HA Chen CY Tang JL Yao M Tsay W Ko BS Wu SJ Huang SY Hsu SC Chen YC Huang YN Chang YC Lee FY Liu MC Liu CW Tseng MH Huang CF Tien HF Distinct clinical and biologic characteristics in adult acute myeloid leukemia bearing the isocitrate dehydrogenase 1 mutation Blood 2010 115 14 2749 54 10.1182/blood-2009-11-253070 20097881

[B52] Boultwood J Perry J Zaman R Fernandez-Santamaria C Littlewood T Kusec R Pellagatti A Wang L Clark RE Wainscoat JS High-density single nucleotide polymorphism array analysis and ASXL1 gene mutation screening in chronic myeloid leukemia during disease progression Leukemia 2010 24 6 1139 45 10.1038/leu.2010.65 20410925

[B53] Pérez B Kosmider O Cassinat B Renneville A Lachenaud J Kaltenbach S Bertrand Y Baruchel A Chomienne C Fontenay M Preudhomme C Cavé H Genetic typing of CBL, ASXL1, RUNX1, TET2 and JAK2 in juvenile myelomonocytic leukaemia reveals a genetic profile distinct from chronic myelomonocytic leukaemia Br J Haematol 2010 151 5 460 8 10.1111/j.1365-2141.2010.08393.x 20955399

[B54] Sugimoto Y Muramatsu H Makishima H Prince C Jankowska AM Yoshida N Xu Y Nishio N Hama A Yagasaki H Takahashi Y Kato K Manabe A Kojima S Maciejewski JP Spectrum of molecular defects in juvenile myelomonocytic leukaemia includes ASXL1 mutations Br J Haematol 2010 150 1 83 7 20408841 10.1111/j.1365-2141.2010.08196.x

[B55] Kosmider O Gelsi-Boyer V Cheok M Grabar S Della-Valle V Picard F Viguié F Quesnel B Beyne-Rauzy O Solary E Vey N Hunault-Berger M Fenaux P Mansat-De Mas V Delabesse E Guardiola P Lacombe C Vainchenker W Preudhomme C Dreyfus F Bernard OA Birnbaum D Fontenay M Groupe Francophone des Myélodysplasies TET2 mutation is an independent favorable prognostic factor in myelodysplastic syndromes (MDSs) Blood 2009 114 15 3285 91 10.1182/blood-2009-04-215814 19666869

[B56] Thol F Friesen I Damm F Yun H Weissinger EM Krauter J Wagner K Chaturvedi A Sharma A Wichmann M Göhring G Schumann C Bug G Ottmann O Hofmann WK Schlegelberger B Heuser M Ganser A Prognostic significance of ASXL1 mutations in patients with myelodysplastic syndromes J Clin Oncol 2011 29 18 2499 506 10.1200/JCO.2010.33.4938 21576631

